# Pendrin inhibitor PDSinh-C01 reverses salt-sensitive hypertension and metabolic acidosis in the 5/6 nephrectomy rat model

**DOI:** 10.1007/s00424-026-03205-9

**Published:** 2026-08-17

**Authors:** Crissy F. Rudolphi, Yoëlle Goos, Usha Musterd-Bhaggoe, Richard van Veghel, Ingrid M. Garrelds, A. H. Jan Danser, Joy Karmakar, Onur Cil, Pedro H. Imenez Silva, Ewout J. Hoorn

**Affiliations:** 1 Department of Internal Medicine, Division Nephrology and Transplantation, Erasmus MC, University Medical Center Rotterdam, Dr. Molewaterplein 40, 3015 GD Rotterdam Rotterdam, the Netherlands; 2 Department of Internal Medicine, Division Vascular Medicine and Pharmacology, Erasmus MC, University Medical Center Rotterdam, Rotterdam, the Netherlands; 3 Department of Pediatrics, University of California San Francisco, San Francisco, CA USA

**Keywords:** 5/6 nephrectomy, Distal nephron, Diuretics, Loop diuretics

## Abstract

Pharmacological inhibition of the chloride-bicarbonate exchanger pendrin with PDSinh-C01 potentiates the diuretic action of furosemide in healthy mice. Because pendrin contributes to acid-base regulation and blood pressure control, it may represent an attractive therapeutic target in chronic kidney disease (CKD). We investigated whether this pendrin inhibitor improves metabolic acidosis and salt-sensitive hypertension in the 5/6 nephrectomy (5/6Nx) model of CKD. Seven-week-old female Sprague Dawley rats underwent 5/6Nx and implantation of telemetry devices for blood pressure monitoring. All animals received a high-salt (2% NaCl) diet and were randomized (n = 7–9/group) to daily subcutaneous injections for three days of vehicle, PDSinh-C01 (10 mg/kg), furosemide (6 mg/kg), or their combination. The 5/6Nx model recapitulated key features of CKD, including reduced eGFR, hypertension, proteinuria, and hyperchloremic metabolic acidosis. Combination therapy produced the greatest reduction in mean arterial pressure (-11 ± 2 mmHg) compared with vehicle (-3 ± 2 mmHg), furosemide (-6 ± 1 mmHg), or pendrin inhibitor alone (-6 ± 2 mmHg). Furosemide and combination therapy increased urine volume at 6h, whereas only combination therapy increased urine volume at 24h. After three days, the pendrin inhibitor alone and combination therapy increased venous bicarbonate concentrations compared with vehicle (24.5 ± 2.1 and 24.3 ± 1.4 vs. 21.4 ± 1.4 mmol/l). In conclusion, PDSinh-C01 combined with furosemide improves salt-sensitive hypertension in CKD, while PDSinh-C01 alone and in combination with furosemide ameliorates metabolic acidosis. These findings support pendrin as a regulator of electrolyte and acid-base balance and as a potential therapeutic target in CKD.

**Supplementary Information:**

The online version contains supplementary material available at https://doi.org/10.1007/s00424-026-03205-9.

## Introduction

Pendrin (encoded by *SLC26A4*) is a chloride-bicarbonate exchanger expressed in the apical plasma membrane of type B and non-A, non-B intercalated cells in the distal nephron [1]. It plays a key role in renal bicarbonate secretion and chloride reabsorption and has therefore been implicated in the regulation of acid-base balance and blood pressure [2, 3]. Pendrin knockout mice do not exhibit fluid-electrolyte or acid-base abnormalities under baseline conditions. However, mice with double knockout of pendrin and the sodium-chloride cotransporter (encoded by *Slc12a3*) develop profound salt wasting [4]. Moreover, acute ablation of pendrin lowers blood pressure in mice [5], and protects animals against aldosterone-induced hypertension [5, 6]. These findings suggest that pendrin may compensate for inhibition of other sodium-chloride reabsorptive transporters and could therefore contribute to the effects of diuretics and the development of diuretic resistance.

To test this hypothesis, the small-molecule pendrin inhibitor PDS_inh_-C01 has previously been evaluated in healthy female mice, with or without the loop diuretic furosemide [7]. The pendrin inhibitor alone had no effect on urine output, osmolality, salt excretion or acid-base balance, whereas its combination with furosemide increased urine output, sodium and chloride excretion. These findings suggest that pendrin inhibition may represent an attractive therapeutic strategy in chronic kidney disease (CKD), a condition characterized by salt-sensitive hypertension and metabolic acidosis, in which diuretics are a cornerstone of therapy [8]. Accordingly, we hypothesized in this study that pendrin inhibition, with or without furosemide, may ameliorate salt-sensitive hypertension and metabolic acidosis in CKD. To address this hypothesis, we evaluated PDS_inh_-C01 alone or in combination with furosemide in the 5/6 nephrectomy (5/6Nx) rat model of CKD.

## Materials and methods

### Animal studies

Female Sprague Dawley rats (Inotiv/Envigo, The Netherlands) were housed socially in individual ventilated cages and were maintained on a 12 h light/dark cycle. Animals received standard food (SDS CRM (P), SDS Special Diets Services, France) and water *ad libitum*.

### Study design

After one week of acclimatization, eight animals were allocated to the sham group, and the remaining animals (*n* = 46) were allocated to the 5/6 nephrectomy group based on body weight to ensure comparable values between groups (age 7 weeks, mean body weight 160 g). The 5/6 nephrectomy model was performed as described previously [9]. Briefly, the first surgery – uninephrectomy of the right kidney – was combined with implantation of a telemetry transmitter in the abdominal aorta for blood pressure monitoring (Data Sciences International, HD-S10). Animals were individually housed from the time of telemetry implantation. After a 10-day recovery period, a resection of two thirds of the left kidney was performed, followed by a 4-week recovery period. Sham animals underwent the same procedures, including telemetry implantation and sham surgeries corresponding to the 5/6 nephrectomy procedures. All surgical procedures were performed under general inhalational isoflurane anesthesia, administered via a precision vaporizer in combination with 20% oxygen (Sigma Delta, Penlon, United Kingdom). Isoflurane concentrations were maintained at 4–5% during induction and 2–3% during maintenance via a face mask. Body temperature was continuously controlled using a heat therapy system (T-pump, Struker, USA) connected to a heating pad. Adequate analgesia was provided using buprenorphine (0.05 mg/kg, subcutaneously). For baseline measurements, animals were allowed to acclimatize for a minimum of 12 h in the metabolic cage followed by a 24 h measurement period where urine was collected using mineral oil (M5904, Sigma Aldrich). Blood was subsequently collected via tail-vein sampling under inhalational isoflurane anesthesia, and baseline blood pressure was recorded continuously for 72 h. Following baseline measurements, all animals were switched from a normal-sodium diet (0.5% NaCl) to a high-salt diet (2% NaCl, both ssniff Spezialdiäten GmbH, Germany) for 11 days to induce salt-sensitive hypertension. During the final three days of the protocol, all 5/6 nephrectomy animals were randomly assigned to one of the treatment groups (*n* = 7–9/group) and received daily subcutaneous injections of vehicle (saline), furosemide (6 mg/kg; F4381, Sigma-Aldrich [10]), PDS_inh_-C01 (10 mg/kg, synthesized in-house [7]), or combination therapy (furosemide plus PDS_inh_-C01). All drugs were dissolved in 10% DMSO in saline; 10% Kolliphor HS was additionally used to solubilize PDS_inh_-C01 [7], and both DMSO and Kolliphor HS were included in the vehicle treatment. All treatments were administered in an unblinded manner. During the final 24 h metabolic cage period, urine was collected at 6, 12 h and 24 h to assess time-dependent diuretic effects. At the end of the study, animals were killed through terminal bleeding via venipuncture of the vena cava under isoflurane anesthesia. After this, organs were collected for additional experiments.

### Biochemical measurements

For measurement of pH, pCO_2_, bicarbonate, sodium, chloride, and potassium, venous blood was collected in lithium heparin tubes and analyzed immediately by an ABL800 blood gas analyzer (Roche, The Netherlands). The remaining blood was collected in either lithium heparin or EDTA tubes. Lithium heparin samples were centrifuged for 10 min at 3,000 rpm at room temperature, and plasma was stored at -80 °C until measurement of osmolality (OSMO Station OM6060, Arkay, Japan) and creatinine (Cobas 8000, C702 module, Roche, The Netherlands). Blood collected in EDTA tubes was immediately placed on ice, centrifuged for 5 min at 5,000 rpm at 4 °C and plasma was stored in -80 °C until measurement of renin and aldosterone. Active plasma renin concentration was determined using an in-house developed assay that measures the rate of angiotensin I generation during incubation with known amounts of angiotensinogen in the presence of angiotensin-converting enzyme, angiotensinase, and serine protease inhibitors (pH 7.4, temperature 37 °C) [11, 12]. Renin concentration was defined as the maximal angiotensin I generation rate (V_max_) at saturating angiotensinogen concentrations and was quantified by radioimmunoassay [11, 12]. Aldosterone was measured using a commercial radioimmunoassay (Beckman Coulter, Immunotech, Czech Republic; sensitivity 12 pg/ml). Urine samples collected during the metabolic cage period were transferred to vacutainers, after which sodium, chloride, potassium, phosphate, creatinine, and protein were measured (Cobas 8000, ISE and C702 module, Roche, The Netherlands). Urine bicarbonate was measured by an ABL800 blood gas analyzer (Roche, The Netherlands) and urine pH was measured using a HI1093B pH electrode (Hanna Instruments, HI5221-02). Urine osmolality was measured using the OSMO Station (OM6060, Arkay, Japan). Estimated glomerular filtration rate (eGFR) was calculated using a validated plasma creatinine and urea-based equation for rats [13]. Free water clearance (Cl_H20_) was calculated using the formula: Cl_H20_ (ml/min) = V x (1-U_Osm_/P_Osm_), where V is urine volume (ml/min), U_Osm_ is urine osmolality (mOsm/kg), and P_Osm_ is plasma osmolality (mOsm/kg). Net acid excretion was calculated using the formula: ammonium excretion + titratable acidity – HCO_3_^−^ excretion (Table S1) [14].

### Immunoblotting

Whole kidneys were homogenized in a buffer containing 0.3 M sucrose, 50 mM Tris-HCl (pH 7.5), 1 mM ethylenediaminetetraacetic acid, 1 mM ethylene glycol tetra-acetic acid, 1 mM dithiothreitol, 1 mM phenylmethylsulfonyl fluoride, 1 mM sodium orthovanadate, 1% (v/v) Triton X-100, 50 mM sodium fluoride and supplemented with phosphatase and protease inhibitor cocktails (Roche). Homogenates were centrifuged at 6,000 rpm for 15 min at 4 °C and supernatants were collected. Total protein concentration was determined using a colorimetric assay (Detergent compatible protein assay, BioRad) and homogenates were denatured in sodium dodecyl sulphate–polyacrylamide gel electrophoresis sample buffer (6x Laemmli buffer) for 10 min at 65 °C. Normalization was done by protein concentration and ~ 40 µg protein of each sample (8 µL) was loaded in a 4–20% Criterion-TGX Precast Gel (Bio-Rad). Samples were electrophoretically separated for 1.5 h in 1x TGS buffer at RT and transferred to a 0.2 μm PVDF membrane (BioRad) using a Trans-Blot Turbo transfer system (BioRad). Next, membranes were blocked with 5% (w/v) non-fat milk or bovine serum albumin [in Tris-buffered saline (TBS) containing 0.1% (v/v) Tween-20] for 2 h. Membranes were incubated overnight at 4 °C with the corresponding diluted primary antibody (Table S2). Afterwards, membranes were washed 5 times 10 min in TBS-T and then incubated for 1 h with horseradish peroxidase conjugated secondary antibody (Table S2). After this incubation, membranes were washed again and signals were quantified by enhanced chemiluminescence reagent (Clarity Western ECL substrate Cat#170–5061, Bio-Rad) using an Amersham 600 system (GE Life Sciences). Densitometry was performed using Image Studio Lite (LI-COR Biosciences, version 5.2).

### Statistical analysis

Data are presented as mean ± standard deviation for normally distributed data and median (interquartile range) for non-normally distributed data. A sample size calculation was performed using a non-central t-distribution for a two-tailed test, powered for the primary comparison between the pendrin inhibitor and vehicle. Secondary analyses examined whether combination therapy with the pendrin inhibitor was superior to furosemide alone or vehicle treatment. A minimum mean arterial blood pressure (MAP) reduction of 15 mmHg was considered clinically relevant. Based on our previously published data in Sprague-Dawley rats and those of others [15], the standard deviation of MAP was estimated at 9.5 mmHg. Assuming an α level of 0.05, a power of 0.8, a minimum of eight animals per group was required. To account for potential losses due to surgery-related morbidity, up to 11 animals per group were planned. In total, 54 animals were enrolled, of which 41 completed the study; attrition was due to surgery-related morbidity. At baseline, differences between sham and 5/6Nx groups were analyzed by Welch’s t-test or Mann-Whitney test. At the end of study, differences between treatment groups for blood and urine parameters were analyzed using one-way ANOVA followed by Tukey’s post hoc multiple-comparisons test or Kruskal-Wallis test followed by Dunn’s multiple comparisons test. Blood pressure data were analyzed using two-way ANOVA with Tukey’s post-hoc multiple-comparisons test. For the hourly blood pressure graph, a smoothed line was generated using locally weighted scatterplot smoothing (LOWESS; 10-point smoothing window). Statistical analyses were performed with GraphPad Prism version 11.0.0 (GraphPad Software, LLC, 2026). A *P-*value < 0.05 was considered statistically significant.

## Results

### Characteristics of the CKD model

**Fig. 1:**
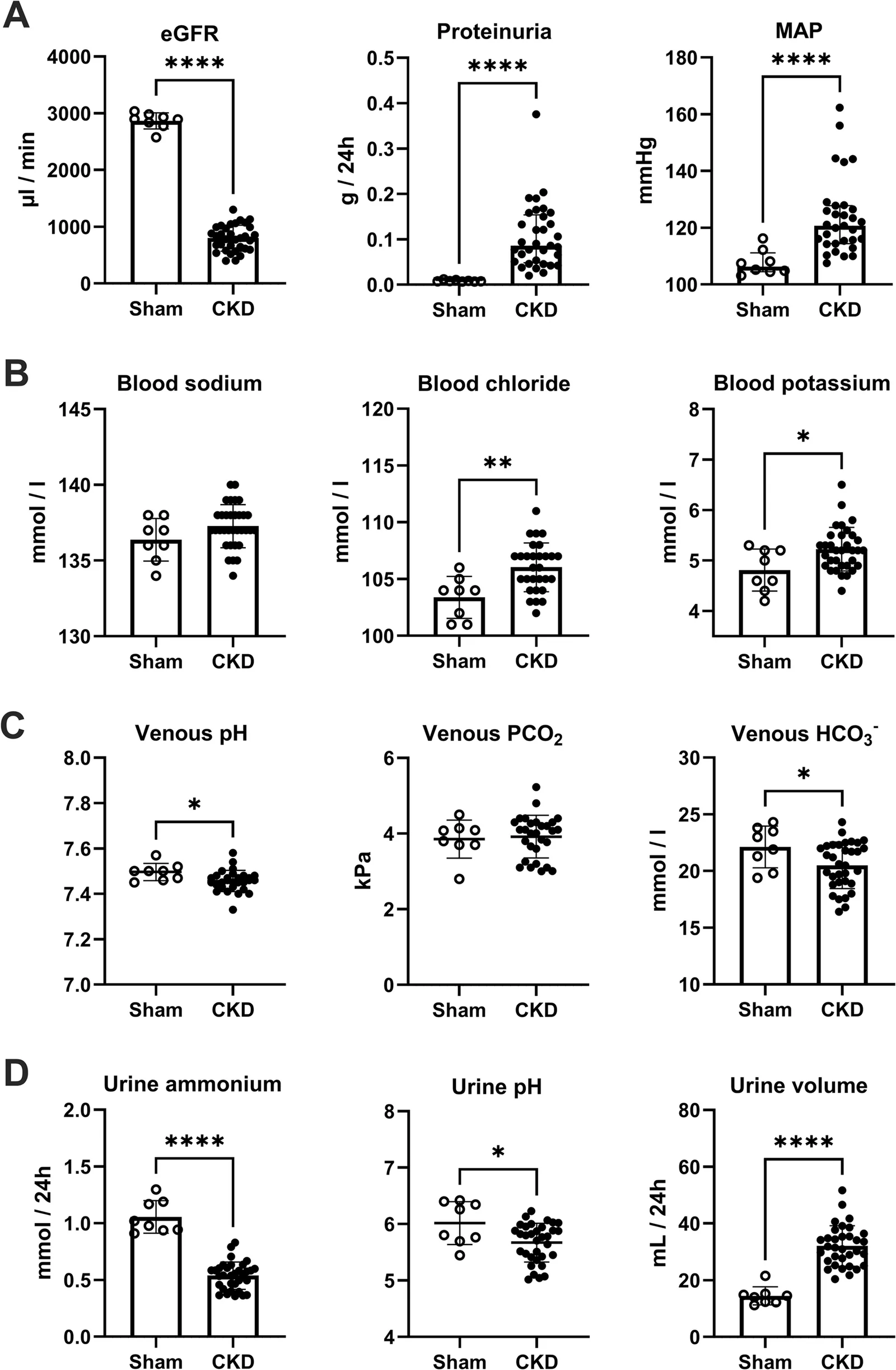
Characterization of the 5/6 nephrectomy model. Baseline characteristics were assessed 4 weeks after completion of the 5/6 nephrectomy and before starting the high-salt diet. (**A)** 5/6 nephrectomy rats (hereafter referred to as CKD, chronic kidney disease) had significantly lower estimated glomerular filtration rate (eGFR), greater proteinuria and higher mean arterial pressure (MAP) compared with sham rats. (**B)** CKD rats had unaltered blood sodium but significantly higher blood chloride and potassium levels than sham rats. (**C)** CKD rats exhibited metabolic acidosis, evidenced by significantly lower venous pH and bicarbonate concentrations, with no change in venous PCO_2_ compared with sham rats. (**D)** CKD rats showed significantly lower urinary ammonium excretion and urine pH compared with sham rats, while urine volume was significantly increased. Data are presented as mean ± SD for normally distributed data and median (interquartile range) for non-normally distributed data. Concentrations are shown in bar graphs, whereas absolute values are shown in dots. Differences between sham (*n* = 8) and CKD (*n* = 33) groups were assessed using Welch’s t-test or Mann-Whitney test. * *P* < 0.05, ** *P* < 0.01, **** *P* < 0.0001

**Fig. 2:**
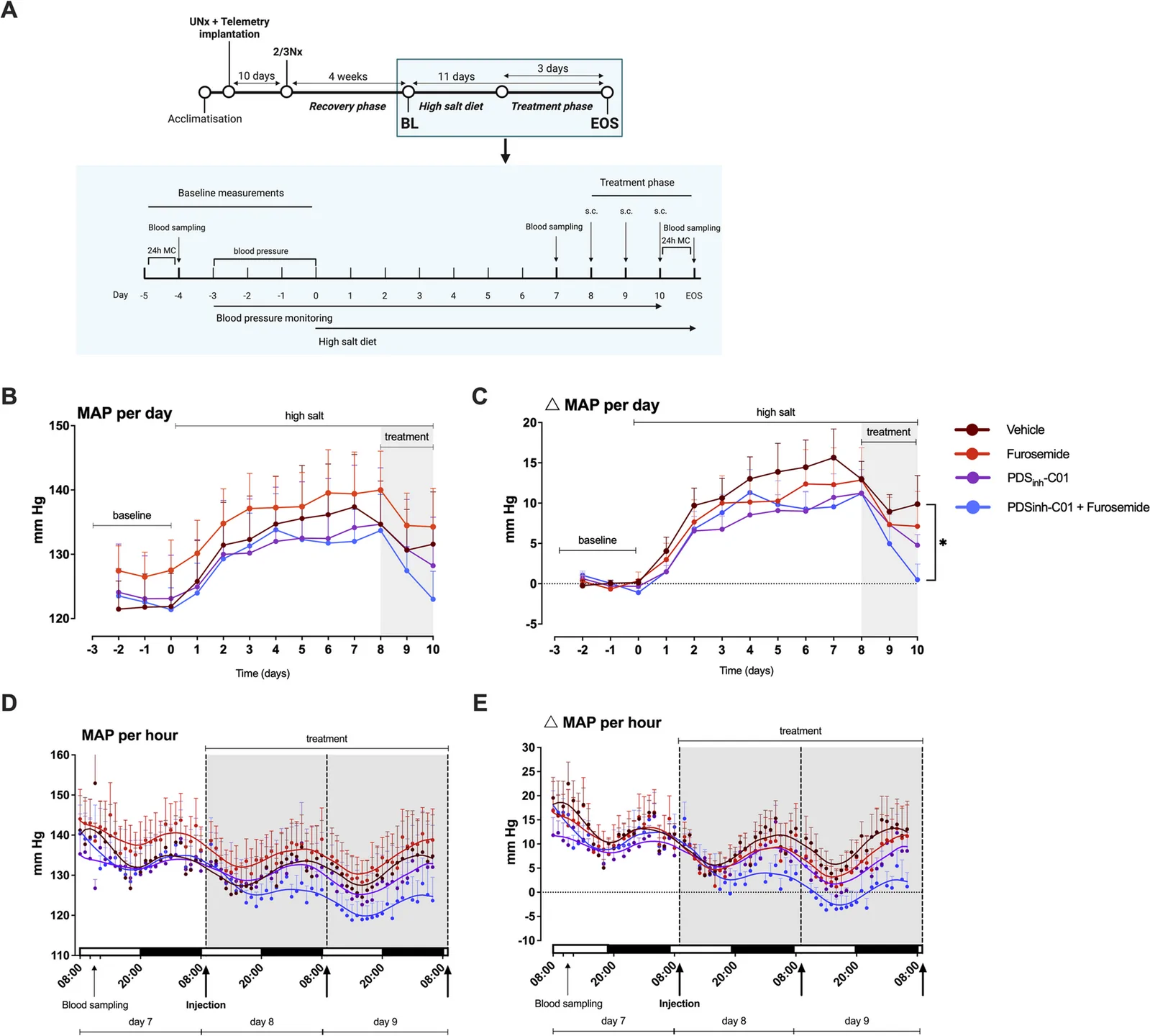
Experimental design and blood pressure response following two days of treatment in 5/6 nephrectomy rats. (**A)** Schematic overview of the study design. Each day is defined as the period from 8:00 am to 8:00 am the following day. Baseline characteristics were assessed 4 weeks after 5/6 nephrectomy. UNx, uninephrectomy; 2/3Nx, 2/3 nephrectomy; BL, baseline. (**B-C)** Change in mean arterial pressure (MAP) and the change in MAP relative to baseline (ΔMAP). Telemetry recordings started on day − 3, with the data for each day representing the mean 24-hour MAP recorded from 8:00 am on the preceding day to 8:00 am on that day. After 8 days on a 2% high-salt diet, female Sprague Dawley rats were treated with vehicle (*n* = 9), furosemide (*n* = 8), PDS_inh_-CO1 (*n* = 7), or furosemide plus PDS_inh_-CO1 (*n* = 7) via daily subcutaneous injections for three days. Blood pressure was recorded during the first two days of treatment (days 8 and 9). Telemetry data were unavailable for one animal due to a blood clot in the telemetry device. For another animal, telemetry data were excluded because corrosion of the device after removal prevented offset calibration. Data are presented as mean ± SEM. Statistical differences were assessed using two-way ANOVA followed by Tukey’s post-hoc multiple-comparisons test. * *P* < 0.05. (**D-E)** MAP and ΔMAP relative to baseline shown per hour

At baseline, before receiving the high-salt diet, 5/6Nx animals exhibited the hallmarks of CKD, including reduced eGFR, proteinuria, and a significantly higher mean arterial pressure (MAP) compared to sham animals (Fig. 1A). Moreover, CKD animals had hyperkalemia, hyperchloremic metabolic acidosis, reduced urinary ammonium excretion, low urine pH and increased urine volume at baseline (Fig. 1B-D). After the baseline measurements, animals were started on a high-salt diet for eleven days, while the treatments were given on days 8–10 (Fig. 2A). Body weight increased normally over the course of the study, with no significant changes between groups (Figure S1).

### Effects on blood pressure

The high-salt diet increased MAP by 11–13 mmHg in the eight days prior to treatment (Fig. 2B-C). After two days of treatment, blood pressure was reduced by vehicle (-3 ± 2 mmHg), furosemide (-6 ± 1 mmHg), the pendrin inhibitor (-6 ± 2 mmHg), and combination therapy (-11 ± 2 mmHg). Combination therapy resulted in a significantly lower MAP compared with vehicle treatment, restoring blood pressure to baseline levels prior to initiation of the high-salt diet. However, MAP remained higher than that observed in sham animals, indicating that the treatment attenuated the salt-induced increase in blood pressure but did not fully normalize blood pressure to sham values. The stronger blood pressure response with combination therapy was also evident in the hourly blood pressure recordings (Fig. 2D-E). Systolic and diastolic blood pressure values followed trends similar to MAP, whereas heart rate and activity were unaffected by any treatment (Figure S2 and S3). Of note, blood pressure declined during the first day of vehicle treatment; this effect may be attributable to blood collection, the initiation of injections, or to the vehicle itself.

### Effects on urinary acid-base and electrolyte parameters

**Fig. 3:**
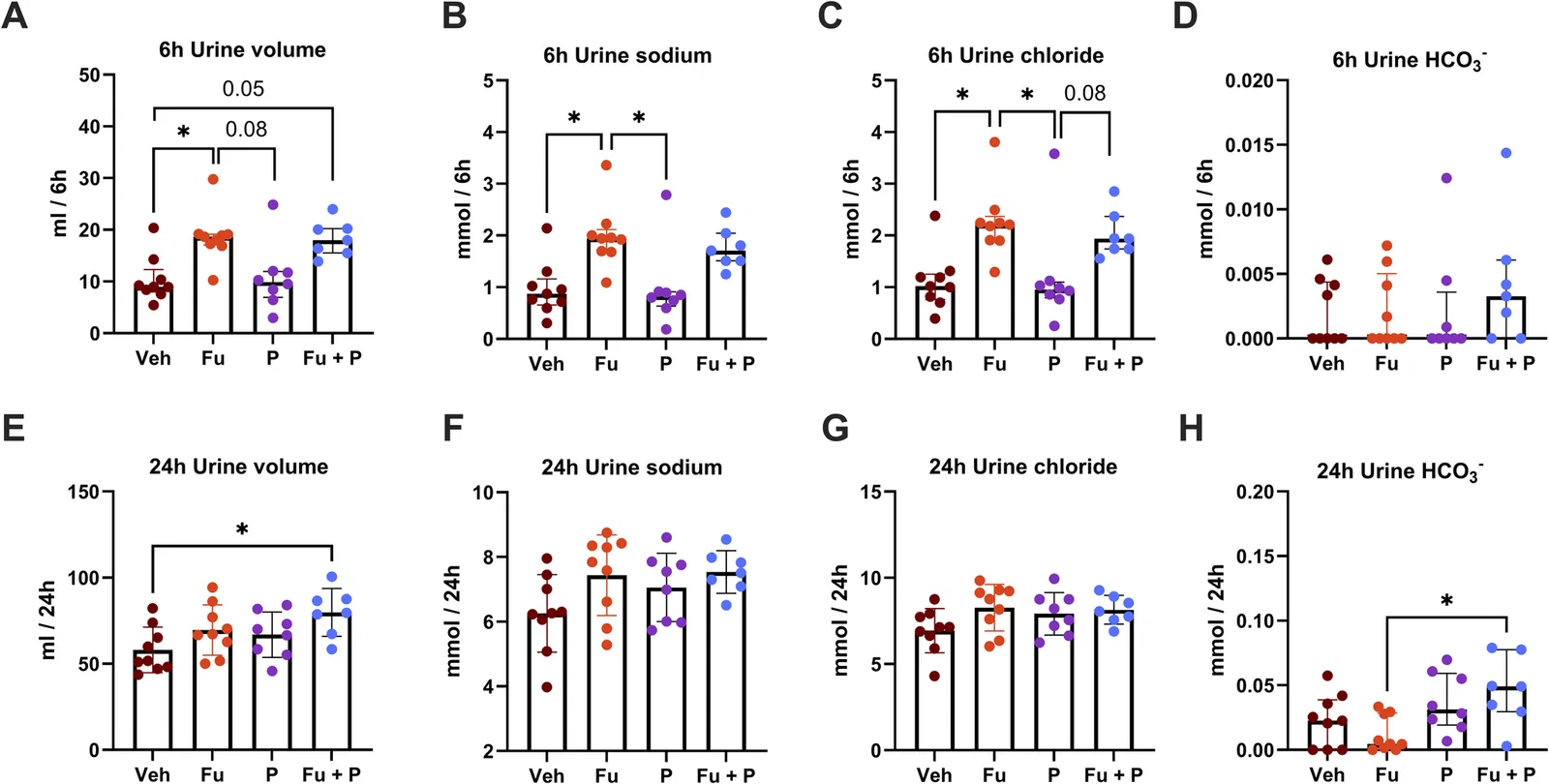
Effects on urine parameters following three days of treatment in 5/6 nephrectomy rats. (**A)** Furosemide (Fu) significantly increased 6 h urine volume compared with vehicle (Veh). (**B-C)** Fu significantly increased 6 h urinary sodium and chloride excretion compared with Veh and PDS_inh_-C01 (P). (**D)** No significant differences in 6 h urinary HCO3^−^ excretion were observed between groups. (**E)** Only combination therapy with Fu + P resulted in a significant increase in 24 h urine volume compared with Veh. (**F-G)** No significant differences in 24 h urinary sodium or chloride excretion were observed between groups. (**H)** Combination therapy of Fu + P significantly increased 24 h urinary HCO3^−^ excretion compared to F alone. All data are presented as mean ± SD for normally distributed data and median (interquartile range) for non-normally distributed data. Veh (*n* = 9), Fu (*n* = 9), P (*n* = 8), Fu + P (*n* = 7). Statistical analyses were performed using one-way ANOVA followed by Tukey’s multiple correction test or Kruskal-Wallis test followed by Dunn’s multiple comparisons test. * *P* < 0.05

On the third day of treatment, animals were placed in metabolic cages for 6- and 24 h urine collections. After 6 h of treatment, furosemide significantly increased urine volume as well as sodium and chloride excretion compared to vehicle. Combination therapy showed the same trend of increased urine volume compared to vehicle, but sodium and chloride excretion did not reach statistical significance (Fig. 3A-C). No changes were observed for 6 h urinary HCO_3_^−^ excretion between the groups (Fig. 3D). After 24 h of treatment, urine volume was significantly increased with combination therapy only (Fig. 3E), with no significant differences in urine sodium and chloride excretion between groups (Fig. 3F-G). Furosemide decreased urinary HCO_3_^−^ excretion, whereas the pendrin inhibitor, alone or combination therapy, increased it; however, only the difference between combination therapy and furosemide was statistically significant (Fig. 3H). The urinary sodium-to-potassium (UNa/K) ratio increased during the first 6 h with both furosemide and combination therapy compared with vehicle, showed no difference during the 6–12 h period, and was higher in the combination group only in the 12–24 h interval, which was also reflected over the full 24 h period; Figure S4 A–D). In addition, no significant differences were observed among groups in urine osmolality, osmole excretion rate, urea excretion, water intake, ammonium excretion, urine pH or net acid excretion (Figure S5 A–G).

### Effects on acid-base and electrolyte parameters in blood

**Fig. 4:**
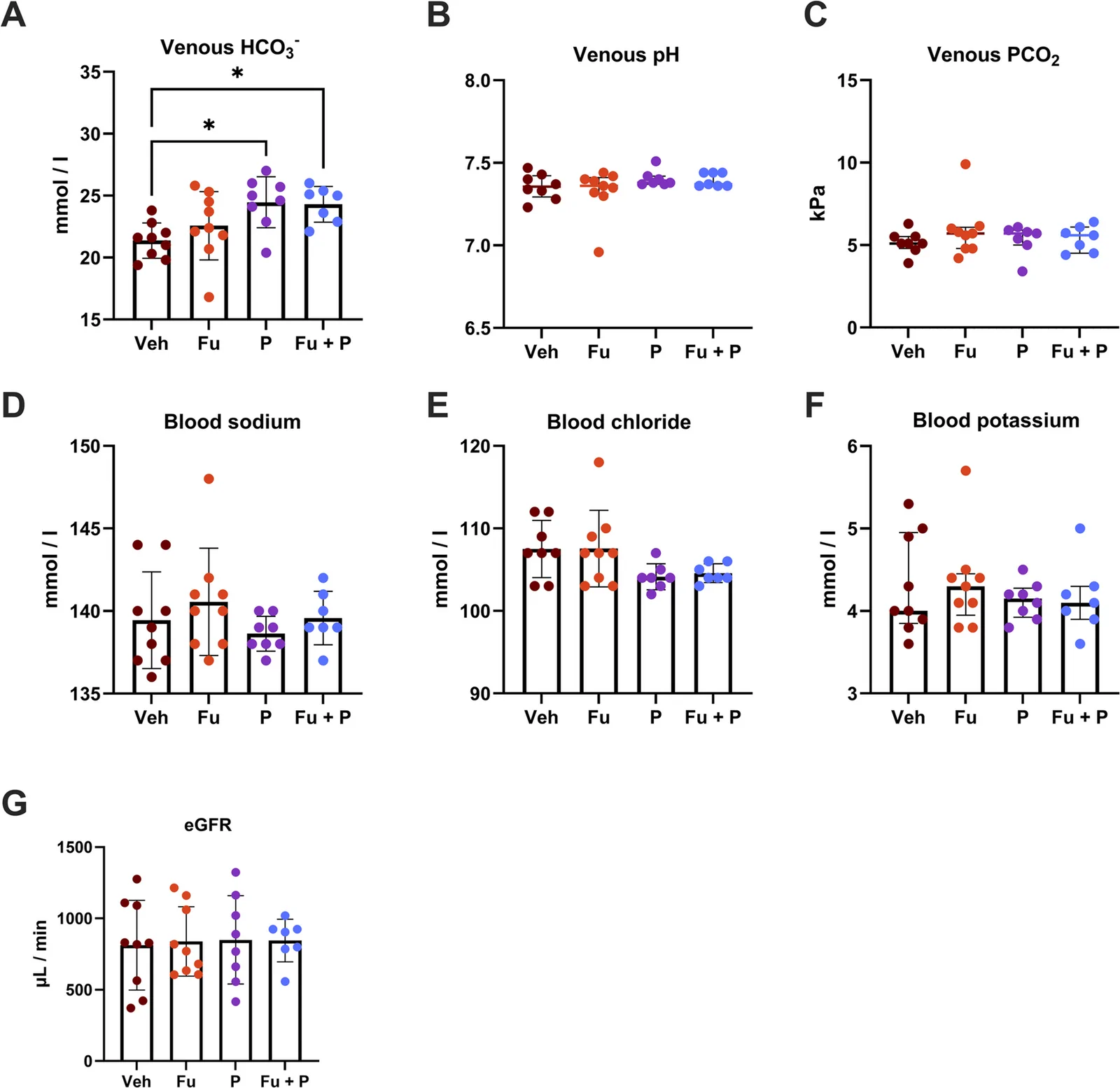
Effects on blood parameters following three days of treatment in 5/6 nephrectomy rats. (**A-C)** No changes were observed for venous pH or PCO_2_ between groups. P and Fu + P significantly increased venous bicarbonate levels compared with Veh. **(D-F)** No significant differences in blood sodium, chloride or potassium concentrations were observed between groups. **(G)** No significant differences for eGFR were observed between groups. All data are presented as mean ± SD for normally distributed data and median (interquartile range) for non-normally distributed data. Concentrations are shown in bar graphs, whereas absolute values are shown in dots. Veh (*n* = 9), Fu (*n* = 9), P (*n* = 8), Fu + P (*n* = 7). Statistical analyses were performed using one-way ANOVA followed by Tukey’s multiple correction test or Kruskal-Wallis test followed by Dunn’s multiple comparisons test. * *P* < 0.05

The blood measurements showed that treatment with the pendrin inhibitor alone and with combination therapy significantly increased venous bicarbonate compared with vehicle (24.5 ± 2.1 and 24.3 ± 1.4 mmol/l vs. 21.4 ± 1.4 mmol/l, respectively; Fig. 4A). No differences were observed for venous pH and pCO_2_ (Fig. 4B-C). None of the treatments significantly affected blood sodium, chloride, or potassium concentrations (Fig. 4D–F) or eGFR (Fig. 4G). Of note, the 5/6 nephrectomy animals were no longer hyperkalemic, possibly due high salt-induced kaliuresis [16]. Finally, plasma renin and aldosterone levels, as well as free water clearance, did not differ significantly across treatment groups (Figure S6).

### Effects on tubular transporter abundance in the kidney

Pendrin was detected in all groups, with no significant differences in abundance observed among groups after three days of treatment (Figure S7). The tubular transporter CFTR, which is functionally coupled with pendrin [17], showed significant decreased abundance in animals treated with both PDS_inh_-C01 and furosemide, as well as a trend towards lower abundance in PDS_inh_-C01 treated animals alone compared to vehicle. No significant differences were observed in the abundance of other proteins involved in tubular transport (NHE3, NBC1, NCC, pNCC, NDCBE and ENaC). However, protein abundances showed substantial variability across groups, likely reflecting the heterogeneity of the model, which limits interpretation of these findings.

## Discussion

Our study demonstrates that in the 5/6 nephrectomy model of CKD, the pendrin inhibitor PDS_inh_-C01 combined with furosemide increases diuresis and ameliorates salt-sensitive hypertension. Moreover, PDS_inh_-C01 alone or in combination with furosemide restores metabolic acidosis. These findings support a role for pendrin in CKD-associated salt-sensitive hypertension and metabolic acidosis, and identify pendrin inhibitors as a potential novel diuretic and anti-hypertensive treatment strategy in CKD.

The blood pressure–lowering effect of combination therapy with the pendrin inhibitor and furosemide is likely attributable to its diuretic action, as evidenced by increased urine volumes at both 6 and 24 h after drug administration. Furosemide alone produced a clear saluretic effect at 6 h, reflected by increased urinary sodium and chloride excretion; combination therapy showed a similar trend but did not reach statistical significance. By 24 h after drug administration, saluretic effects were no longer evident with either furosemide alone or combination therapy, suggesting establishment of a new steady state. In contrast, enhanced diuresis persisted at 24 h only in the combination therapy group, suggesting that pendrin inhibition prevented compensatory responses after the effects of furosemide subsided. This interpretation is consistent with previous findings in healthy mice showing that pendrin inhibition alone did not increase urine output but potentiated the diuretic effect of furosemide [7]. Although enhanced diuresis with combination therapy was expected to be accompanied by body weight reduction and increased plasma renin levels, neither was observed, possibly due to the high-salt diet and the low-renin characteristics of the remnant kidney model [18].

The other notable finding in this study was that the pendrin inhibitor, both alone and combined with furosemide, increased plasma bicarbonate levels and corrected metabolic acidosis. An increase in plasma bicarbonate, or the development of overt metabolic alkalosis, is commonly observed during diuretic therapy and is attributed to enhanced proximal and distal tubular bicarbonate reabsorption driven by angiotensin II, hypochloremia, and hypokalemia [19, 20]. However, in the present study, furosemide alone did not significantly increase plasma bicarbonate levels, suggesting that the effect of the pendrin inhibitor occurred through a different mechanism. Specifically, inhibition of pendrin may have reduced bicarbonate secretion in type B intercalated cells, thereby increasing plasma bicarbonate levels. The small increase in urinary bicarbonate excretion observed after three days of combination therapy, compared to furosemide alone, likely reflects greater filtration and excretion of bicarbonate under improved acid-base status.

Pendrin activity is strongly modulated by acid-base status, dietary chloride intake and aldosterone [21]. Our experimental model incorporates alterations in all three factors: metabolic acidosis, high NaCl intake, and elevated aldosterone levels [22]. Metabolic acidosis and high chloride intake are expected to inhibit pendrin activity, whereas increased aldosterone would stimulate it. The consistent and significant effects of pendrin inhibition on blood pressure and acid-base balance indicate that pendrin remains functionally active in the 5/6 nephrectomy model despite opposing regulatory influences.

We have previously demonstrated increased ENaC activity in the 5/6Nx model [22], and pendrin has been shown to enhance ENaC function by modulating luminal bicarbonate concentrations [23]. Combination therapy with the pendrin inhibitor and furosemide increased the urinary Na/K ratio at 24 h, consistent with reduced ENaC-mediated sodium reabsorption, although this was not reflected by increased ENaC protein abundance following any of the treatments. The parallel increase in urine volume suggests sustained distal natriuresis, which likely contributed to the greater reduction in blood pressure observed with combination therapy. These findings support the concept that pendrin inhibition attenuates ENaC-dependent sodium retention, particularly under conditions of high distal sodium delivery during loop diuretic treatment [24]. Moreover, the observed reduction in CFTR abundance is consistent with the close functional interaction between CFTR and pendrin described previously [17], although the present data do not establish a causal relationship between pendrin inhibition and CFTR downregulation.

A major strength of this study is that it represents the first application of the pendrin inhibitor PDS_inh_-C01 in a CKD model. Furthermore, this is the first report of PDS_inh_-C01 use in rats, demonstrating that the compound is efficacious across species. Nevertheless, several limitations should be acknowledged. First, we did not perform pharmacokinetic measurements of PDS_inh_-C01 following subcutaneous administration, and therefore cannot determine the duration of systemic exposure or directly correlate plasma drug concentrations with the observed blood pressure responses. Future studies incorporating pharmacokinetic and pharmacodynamic analyses will be required to define the exposure-response relationship and duration of target engagement. Second, we cannot exclude potential pleiotropic effects of PDS_inh_-C01, although our findings are consistent with pendrin inhibition. Third, larger quantities of PDS_inh_-C01 were required compared to previous studies, because the compound was tested in rats rather than mice. As PDS_inh_-C01 is not commercially available and must be synthesized in-house, production is costly and labor-intensive. Consequently, we were only able to evaluate the short-term effects of PDS_inh_-C01 in female rats. Previous research with the PDS_inh_-C01 compound was also limited to females [7]. Future studies should therefore assess the longer-term effects of PDS_inh−_C01 and include male rats. Fourth, this was a proof-of-principle study and was not designed to elucidate the underlying mechanisms of the anti-hypertensive and alkalinizing effects of PDS_inh_-C01. To avoid potential interference with telemetric blood pressure recordings, metabolic cage studies were limited to the final 24 h on the third day of treatment. At this time, a new steady state may have been reached and early changes in sodium and electrolyte excretion during the first hours or days of treatment may not have been captured. Future studies should therefore include cumulative electrolyte and acid-base balances measurements, including measures of pendrin activity such as urinary bicarbonate excretion. Moreover, longer periods of pendrin inhibition should be taken into account to investigate any potential safety concerns or side-effects of the drug. Such studies could also take advantage of additional pendrin inhibitors that have been developed more recently and could add available pendrin-deficient animals [25, 26].

In conclusion, pendrin inhibition combined with loop diuretic therapy improves salt-sensitive hypertension in a rat model of CKD, while the pendrin inhibitor PDS_inh_-C01 alone and in combination with furosemide ameliorates metabolic acidosis.

## Supplementary Information

Below is the link to the electronic supplementary material.

Supplementary Material 1 (DOCX 3.00 MB)

## Data Availability

All data supporting the findings of this study are available within the paper and its Supplementary Information.

## References

[1] Royaux IE, Wall SM, Karniski LP, Everett LA, Suzuki K, Knepper MA, Green ED (2001) Pendrin, encoded by the Pendred syndrome gene, resides in the apical region of renal intercalated cells and mediates bicarbonate secretion. Proc Natl Acad Sci U S A 98:4221–4226 11274445 10.1073/pnas.071516798 PMC31206

[2] Brazier F, Corniere N, Picard N, Chambrey R, Eladari D (2024) Pendrin: linking acid base to blood pressure. Pflugers Arch 476:533–543 38110744 10.1007/s00424-023-02897-7

[3] Wall SM (2016) The role of pendrin in blood pressure regulation. Am J Physiol Ren Physiol 310:F193–203 10.1152/ajprenal.00400.2015 PMC4888565 26538443

[4] Soleimani M, Barone S, Xu J, Shull GE, Siddiqui F, Zahedi K, Amlal H (2012) Double knockout of pendrin and Na-Cl cotransporter (NCC) causes severe salt wasting, volume depletion, and renal failure. Proc Natl Acad Sci U S A 109:13368–13373 22847418 10.1073/pnas.1202671109 PMC3421168

[5] Trepiccione F, Soukaseum C, Baudrie V, Kumai Y, Teulon J, Villoutreix B, Cornière N, Wangemann P, Griffith AJ, Byung Choi Y, Hadchouel J, Chambrey R, Eladari D (2017) Acute genetic ablation of pendrin lowers blood pressure in mice. Nephrol Dial Transpl 32:1137–1145 10.1093/ndt/gfw393 PMC5837383 28064162

[6] Verlander JW, Hassell KA, Royaux IE, Glapion DM, Wang ME, Everett LA, Green ED, Wall SM (2003) Deoxycorticosterone upregulates PDS (Slc26a4) in mouse kidney: role of pendrin in mineralocorticoid-induced hypertension. Hypertension 42:356–362 12925556 10.1161/01.HYP.0000088321.67254.B7

[7] Cil O, Haggie PM, Phuan PW, Tan JA, Verkman AS (2016) Small-Molecule Inhibitors of Pendrin Potentiate the Diuretic Action of Furosemide. J Am Soc Nephrol 27:3706–3714 27153921 10.1681/ASN.2015121312 PMC5118489

[8] Agarwal R, Verma A, Georgianos PI (2025) Diuretics in patients with chronic kidney disease. Nat Rev Nephrol 21:264–278 39775051 10.1038/s41581-024-00918-x

[9] Wei KY, Gritter M, Danser AHJ, Vogt L, de Borst MH, Rotmans JI, Imenez Silva PH, Hoorn EJ (2024) 5/6 Nephrectomy Impairs Acute Kaliuretic Responses and Predisposes to Postprandial Hyperkalemia. Am J Physiol Ren Physiol 327:F1005–F1012 10.1152/ajprenal.00195.2024 39417828

[10] Frindt G, Yang L, Uchida S, Weinstein AM, Palmer LG (2017) Responses of distal nephron Na(+) transporters to acute volume depletion and hyperkalemia. Am J Physiol Ren Physiol 313:F62–F73 10.1152/ajprenal.00668.2016 PMC5538837 28356292

[11] Danser AH, van Kats JP, Admiraal PJ, Derkx FH, Lamers JM, Verdouw PD, Saxena PR, Schalekamp MA (1994) Cardiac renin and angiotensins. Uptake from plasma versus in situ synthesis. Hypertension 24:37–48 8021006 10.1161/01.hyp.24.1.37

[12] Ye D, Cruz-López EO, Veghel RV, Garrelds IM, Kasper A, Wassarman K, Tu HC, Zlatev I, Danser AHJ (2024) Counteracting Angiotensinogen Small-Interfering RNA-Mediated Antihypertensive Effects With REVERSIR. Hypertension 81:1491–1499 38690653 10.1161/HYPERTENSIONAHA.124.22878 PMC11177597

[13] Besseling PJ, Pieters TT, Nguyen ITN, de Bree PM, Willekes N, Dijk AH, Bovée DM, Hoorn EJ, Rookmaaker MB, Gerritsen KG, Verhaar MC, Gremmels H, Joles JA (2021) A plasma creatinine- and urea-based equation to estimate glomerular filtration rate in rats. Am J Physiol Ren Physiol 320:F518–F524 10.1152/ajprenal.00656.2020 33522412

[14] Ferraro PM, Taylor EN, Asplin JR, Curhan GC (2023) Associations between Net Gastrointestinal Alkali Absorption, 24-Hour Urine Lithogenic Factors, and Kidney Stones. Clin J Am Soc Nephrol 18:1068–1074 37256914 10.2215/CJN.0000000000000195 PMC10564372

[15] Dayton A, Exner EC, Bukowy JD, Stodola TJ, Kurth T, Skelton M, Greene AS, Cowley AW Jr (2016) Breaking the Cycle: Estrous Variation Does Not Require Increased Sample Size in the Study of Female Rats. Hypertension 68:1139–1144 27672030 10.1161/HYPERTENSIONAHA.116.08207 PMC5104284

[16] Ellison DH, Velazquez H, Wright FS (1989) Adaptation of the distal convoluted tubule of the rat. Structural and functional effects of dietary salt intake and chronic diuretic infusion. J Clin Invest 83:113–126 2910903 10.1172/JCI113847 PMC303651

[17] Berg P, Jeppesen M, Leipziger J (2021) Cystic fibrosis in the kidney: new lessons from impaired renal HCO3- excretion. Curr Opin Nephrol Hypertens 30:437–443 34027905 10.1097/MNH.0000000000000725

[18] Pupilli C, Chevalier RL, Carey RM, Gomez RA (1992) Distribution and content of renin and renin mRNA in remnant kidney of adult rat. Am J Physiol 263:F731–738 1415744 10.1152/ajprenal.1992.263.4.F731

[19] Emmett M (2020) Metabolic Alkalosis: A Brief Pathophysiologic Review. Clin J Am Soc Nephrol 15:1848–1856 32586924 10.2215/CJN.16041219 PMC7769018

[20] Wesson DE (1989) Augmented bicarbonate reabsorption by both the proximal and distal nephron maintains chloride-deplete metabolic alkalosis in rats. J Clin Invest 84:1460–1469 2808701 10.1172/JCI114321 PMC304010

[21] Wall SM, Verlander JW, Romero CA (2020) The Renal Physiology of Pendrin-Positive Intercalated Cells. Physiol Rev 100:1119–1147 32347156 10.1152/physrev.00011.2019 PMC7474261

[22] Bovee DM, Uijl E, Severs D, Rubio-Beltran E, van Veghel R, van den Maassen A, Joles JA, Zietse R, Cuevas CA, Danser AHJ, Hoorn EJ (2021) Dietary salt modifies the blood pressure response to renin-angiotensin inhibition in experimental chronic kidney disease. Am J Physiol Ren Physiol 320:F654–F668 10.1152/ajprenal.00603.2020 33586496

[23] Pech V, Pham TD, Hong S, Weinstein AM, Spencer KB, Duke BJ, Walp E, Kim YH, Sutliff RL, Bao HF, Eaton DC, Wall SM (2010) Pendrin modulates ENaC function by changing luminal HCO3. J Am Soc Nephrol 21:1928–1941 20966128 10.1681/ASN.2009121257 PMC3014007

[24] Wagner CA (2016) Pendrin-A New Target for Diuretic Therapy? J Am Soc Nephrol 27:3499–3501 27516234 10.1681/ASN.2016070720 PMC5118499

[25] Choi JS, Shin MH, Oh GE, Song DN, NamKung W, Han GH, Choi JY, Park MS (2025) Pendrin inhibition is associated with protective effect of prone positioning in a ventilator-induced lung injury mouse model. Sci Rep 15:40926 41266416 10.1038/s41598-025-24241-y PMC12635137

[26] Master RJ, Karmakar J, Haggie PM, Anthony-Tan J, Chu T, Verkman AS, Anderson MO, Cil O (2024) High potency 3-carboxy-2-methylbenzofuran pendrin inhibitors as novel diuretics. Eur J Med Chem 283:117133 39642691 10.1016/j.ejmech.2024.117133 PMC13011504

