# Supplementary material for: Pendrin inhibitor PDSinh-C01 reverses salt-sensitive hypertension and metabolic acidosis in the 5/6 nephrectomy rat model
Source: Pflugers Arch. 2026 Aug 17;478(9):75. doi: 10.1007/s00424-026-03205-9 (PMC13481392; doi:10.1007/s00424-026-03205-9)
Supplement: Supplementary file 1 [file 424_2026_3205_MOESM1_ESM.docx]

**Supplementary Material**

**Pendrin inhibitor PDSinh-CO1 reverses salt-sensitive hypertension**

**and metabolic acidosis in the** **5/6 nephrectomy rat model**

Crissy F. Rudolphi, Yoëlle Goos, Usha Musterd-Bhaggoe, Richard van Veghel,

Ingrid M. Garrelds, A.H. Jan Danser, Joy Karmakar, Onur Cil,

Pedro H. Imenez Silva, Ewout J. Hoorn

**Table of contents**

- **Table S1.** Equations used in this study.
- **Table S2.** Antibodies used for immunoblotting.
- **Figure S1.** Body weight over the course of the study.
- **Figure S2.** Systolic and diastolic blood pressure, heart rate, and activity following two days of treatment in 5/6 nephrectomy rats.
- **Figure S3.** Systolic and diastolic blood pressure per hour following two days of treatment in 5/6 nephrectomy rats.
- **Figure S4.** Urine Na/K ratio following three days of treatment in 5/6 nephrectomy rats.
- **Figure S5.** Urine osmolality, urine osmole, urea and ammonium excretion, urine pH and net acid excretion following three days of treatment in 5/6 nephrectomy rats.
- **Figure S6.** Plasma renin and aldosterone, and free water clearance following three days of treatment in 5/6 nephrectomy rats.
- **Figure S7.** Tubular transporter abundance in the kidney following three days of treatment in 5/6 nephrectomy rats.
- **ARRIVE guidelines**

**Table S1.** Equations used in this study.

| **Titratable acidity** | $\left[ 0.8*{\mathrm{UP}-10}^{\left( UpH-6.8 \right)}*\frac{\mathrm{UP}}{\left( {1+10}^{\left( UpH-6.8 \right)} \right)} \right]+$  $\left[ 0.996*{\mathrm{UCrea}-10}^{\left( UpH-4.92 \right)}*\frac{\mathrm{UCrea}}{\left( {1+10}^{\left( UpH-4.92 \right)} \right)} \right]$ |
| --- | --- |
| **Renal net acid excretion** | Urinary ammonium + titratable acidity – measured urinary bicarbonate (all in mmol/24h) |

U before an abbreviation indicates Urine. Crea, creatinine; P, phosphate; K, potassium; Ca, calcium; Mg, magnesium. Adjusted from [1].

**Table S2.** Antibodies used for immunoblotting.

| **Primary antibody** | **Source** | **Dilution** | **Species** |
| --- | --- | --- | --- |
| Pendrin | C. Wagner (D115) | 1:500 | Rabbit |
| NHE3 | StressMarq (#SPC-400D) | 1:1000 | Rabbit |
| NBC1 (SLC4A4) | Proteintech (Cat #11885-1-AP) | 1:1000 | Rabbit |
| tNCC | Millipore (AB3553) | 1:1000 | Rabbit |
| pT53-NCC | R.Fenton | 1:500 | Rabbit |
| NDCBE (SLC4A8) | Christian Hubner (K1P1) | 1:1000 | Rabbit |
| **α-**ENaC | StressMarq (#SPC-403) | 1:1000 | Rabbit |
| γ-ENaC | StressMarq (#SPC-405) | 1:3000 | Rabbit |
| CFTR | Alomone Labs (ACL-006) | 1:200 | Rabbit |
| GAPDH | Proteintech (Cat #10494-1-AP) | 1:10000 | Rabbit |
| **Secondary antibody** | **Source** | **Dilution** | **Species** |
| Goat Anti-Rabbit IgG (H+L) | BioRad (Cat #1706515) | 1:5000 | Goat |

**Figure S1.** Body weight over the course of the study.

**Legend:** MC, metabolic cage. All data are presented as mean ± SD. Vehicle (n = 9), Furosemide (n = 9), PDS_inh_-C01 (n = 8), PDS_inh_-C01 + Furosemide (n = 7).

**Figure S2.** Systolic and diastolic blood pressure, heart rate, and activity following two days of treatment in 5/6 nephrectomy rats.

**Legend:** After 8 days on a 2% high-salt diet, female Sprague Dawley rats were treated with vehicle (n = 9), furosemide (n = 8), PDS_inh_-CO1 (n = 7), or furosemide plus PDS_inh_-CO1 (n = 7) via daily subcutaneous injections for three days. Telemetry data were unavailable for one animal due to a blood clot in the telemetry device. For another animal, telemetry data were excluded because corrosion of the device after removal prevented offset calibration. Data are presented as mean ± SEM. Statistical differences were assessed using two-way ANOVA followed by Tukey’s post-hoc multiple-comparisons test.

**Figure S3.** Systolic and diastolic blood pressure per hour following two days of treatment in 5/6 nephrectomy rats.

**Legend:** Absolute values and changes (Δ) in systolic blood pressure (SBP) and diastolic blood pressure (DBP) relative to baseline, shown per hour. Data are presented as mean ± SEM.

**Figure S4.** Urine Na/K ratio following three days of treatment in 5/6 nephrectomy rats.

**Legend:** 24-hour urine was collected following three days of treatment. Urinary sodium‑to‑potassium ratios (UNa/K) are shown for urine collected during the first 0–6 h **(A)**, 6–12 h **(B**), 12–24 h **(C)**, and over the entire 24‑h collection period **(D)**. Veh, vehicle; Fu, furosemide; P, PDS_inh_‑C01. Data are presented as mean ± SD for normally distributed data or median (interquartile range) for non‑normally distributed data. Group sizes were: Veh (n = 9), Fu (n = 9), P (n = 8), Fu + P (n = 7). Statistical analyses were performed using one‑way ANOVA with Tukey’s multiple‑comparisons test or Kruskal–Wallis test with Dunn’s post hoc test. **P* < 0.05, ***P* < 0.01, ****P* < 0.001.

**Figure S5.** Urine osmolality, urine osmole, urea and ammonium excretion, and urine pH following three days of treatment in 5/6 nephrectomy rats.

**Legend: (A-E)** 24h values of urine osmolality, urine osmole excretion, urine urea and water intake. **(F)** pH measured in the 12-24h collected urine. (**G**) Calculated total 24h net acid excretion. Veh, vehicle; Fu, furosemide; P, PDS_inh_-C01. Data are presented as mean ± SD for normally-distributed data and median (interquartile range) for non-normally distributed data. Veh (n = 9), Fu (n = 9), P (n = 8), Fu + P (n = 7). Statistical analyses were performed using one-way ANOVA followed by Tukey’s multiple correction test or Kruskal-Wallis test followed by Dunn’s multiple comparisons test.

**Figure S6.** Plasma renin and aldosterone, and free water clearance following three days of treatment in 5/6 nephrectomy rats.

**Legend:** Veh, vehicle; Fu, furosemide; P, PDS_inh_-C01. All data are presented as mean ± SD. Veh (n = 9), Fu (n = 9), P (n = 8), Fu + P (n = 7). Statistical analyses were performed using one-way ANOVA followed by Tukey’s multiple correction test.

**Figure S7.** Tubular transporter abundance in the kidney following three days of treatment in 5/6 nephrectomy rats.

**Legend (A)** Immunoblots performed on 5/6 nephrectomy animals. One sham animal was used as a reference control sample. **(B)** Corresponding densitometry of the immunoblots performed on whole kidney homogenates from 5/6 nephrectomy rats. Band intensities were normalized to GAPDH and to the mean intensity of the vehicle treated group defined as 1.0. Values displayed under respective blots are median (interquartile range) (n=7-9 per group). Veh, vehicle; Fu, furosemide; P, PDS_inh-_CO1; Fu + P, furosemide and PDS_inh-_CO1. GAPDH was used as a housekeeping gene. One-way ANOVA followed by Tukey’s multiple comparisons test for normally distributed data or Kruskal-Wallis test followed by Dunn’s multiple correction test for non-normally distributed data were used to examine statistical differences. **P*< 0.05.

**References**

1. Ferraro PM, Taylor EN, Asplin JR, Curhan GC (2023) Associations between Net Gastrointestinal Alkali Absorption, 24-Hour Urine Lithogenic Factors, and Kidney Stones. Clin J Am Soc Nephrol 18:1068-1074
